# Species composition, infection rate and feeding preference of *Anopheles* mosquito species (Diptera: Culicidae) in the west Amhara Region, northwest Ethiopia

**DOI:** 10.1371/journal.pone.0307063

**Published:** 2024-07-25

**Authors:** Mulat Yimer, Mastewal Worku, Tsehaynesh Gebreyesus, Addisu Melese, Getaneh Alemu, Michael Alehegne, Taye Zeru, Amare Desta, Tesfa Demilie, Abraham Amsalu, Damtie Lankir, Simeneh Ayalew, Fikirte Estifanos, Belay Bezabih, Banchamlak Tegegne

**Affiliations:** 1 Department of Medical Laboratory Sciences, College of Medicine and Health Sciences, Bahir Dar University, Bahir Dar, Ethiopia; 2 Amhara Public Health Institute, Bahir Dar, Ethiopia; Federal University of Agriculture, Abeokuta, NIGERIA

## Abstract

**Background:**

Reports showed that Amhara Region is accounting for 31% of Ethiopia`s malaria burden. Reports also depicted that despite the existing malaria vector control tools implemented by the regional government, currently there is an increment in malaria prevalence in the region. This might be due to lack of entomological monitoring and comprehensive information on the prevailing species composition, infection rate and feeding presences of *An*. mosquito species in the study areas. Therefore, this study aimed to address this information.

**Methods:**

A community-based cross-sectional study was conducted from 18 February to 30 March 2023 at three districts of the west Amhara Region. For this, six CDC light traps (three indoor and three outdoor) were used to collect adult female *An*. mosquito species. In this study, 261 *An*. mosquito species comprising seven species were collected. Their morphological identification and abdominal status determination were carried out using standard morphological identification keys under a dissection microscope. While their infection rates and blood meal sources were determined, using circumsporozoite protein-ELISA and blood meal-ELISA based on the standard protocol. Data were entered to SPSS versions 20 for analysis and p- value < 0.05 was considered as statistically significant.

**Results:**

In our study, total of 261 *An*. mosquito species were identified. *An*. *demeilloni* was the most abundant species accounted for 112(42.9%) of all captures. It was also the most predominant species at Ayehu-Guagusa followed by Jabitehnan districts. *An*. *demeilloni* and *An*. *cinereus* altogether accounting for infection rate of 3(1.1%) for *P*. *vivax* parasite. Furthermore, our study showed that more than 50% of *An*. mosquito species collected fed on cattle blood showing shifting of feeding behaviors.

**Conclusions:**

Our study depicted that *An*. *demeilloni* and *An*. *cinereus* were the most abundant species and infected with *P*. *vivax* parasite. Therefore, further comprehensive study should be done in the future.

## Background

Although over 3,500 mosquito species are found globally, more than 400 of them are *An*. mosquitoes, of which an average of 60 species are responsible for malaria transmission “[1]”. In Africa, increment in longevity and strong human biting preference made high malaria transmission “[2]” and according to this report, *An*. *gambiae* complex and the funestus subgroups are the most essential malaria vectors in Africa.

Though Ethiopia has implemented malaria vector control tools like indoor residual spraying (IRS), insecticide-treated nets (ITNs), and as an additional tool larval source management (LSM), yet malaria prevalence increased enormously “[3]”. For instances, according to the National Malaria Guideline of Ethiopia “[4]” report, Ethiopia is one of the malaria- endemic countries in Africa and the majority of malaria cases are due to *Plasmodium falciparum* (*P*. *falciparum) 70%*, *P*. *vivax* 30% and *P*. *ovale* reported rarely. Even if the specific favorable altitude(s) at which malaria transmission occurred have been not yet described the country, those below and above 2000 meters above sea level have been reported by scholars “[4, 5]”. Despite this scenario, transmission is seasonal with major (September to December) and minor (from February to March) “[6]”.

Reports showed that Amhara Region is one of the most malaria prevalent region in Ethiopia and accounting for 31% of Ethiopia`s malaria burden “[3, 7]”. Even if several factors might contribute for such increment “[8, 9]”, most importantly, it might be due lack of entomological monitoring “[7]” and drastic altitudinal range from 506 meters at Blue Nile Gorge to 4533 meters at Ras Dejen mountain that create favorable environment for malaria vectors breeding site “[10]”. These and other factors halted the malaria elimination plan that will be achieved by the Ethiopian Ministry of Health by 2025.

In Ethiopia, according to WHO`s “[3]” malaria report, *An*. mosquito species namely, *An*. *arabiensis*, *An*. *funestus*, *An*. *pharoensis*, and *An*. *nili* are malaria vectors (*An*. *arabiensis* is the min vector and *An*. *funestus*, *An*. *pharoensis*, and *An*. *nili* are secondary vectors). However, different pocket studies in Ethiopia “[11–14]”, reported different vector species composition, infection rate and blood meal sources. Likewise, in Amhara Region, even if there has been lack of entomological monitoring, comprehensive information on species composition, infection rate and blood meal sources, some pocket studies “[15–19]”, reported different vector species composition, infection rate and blood meal sources. Due to this, there is lack of comprehensive information on the prevailing species composition, infection rate and feeding preferences in the study areas. This hampered for selecting, designing and implementing appropriate intervention methods to intercept human–vector contact. Therefore, this study aimed to address this information in west Amhara Region northwest Ethiopia.

## Materials and methods

### Study design, period and area

**Fig 1:**
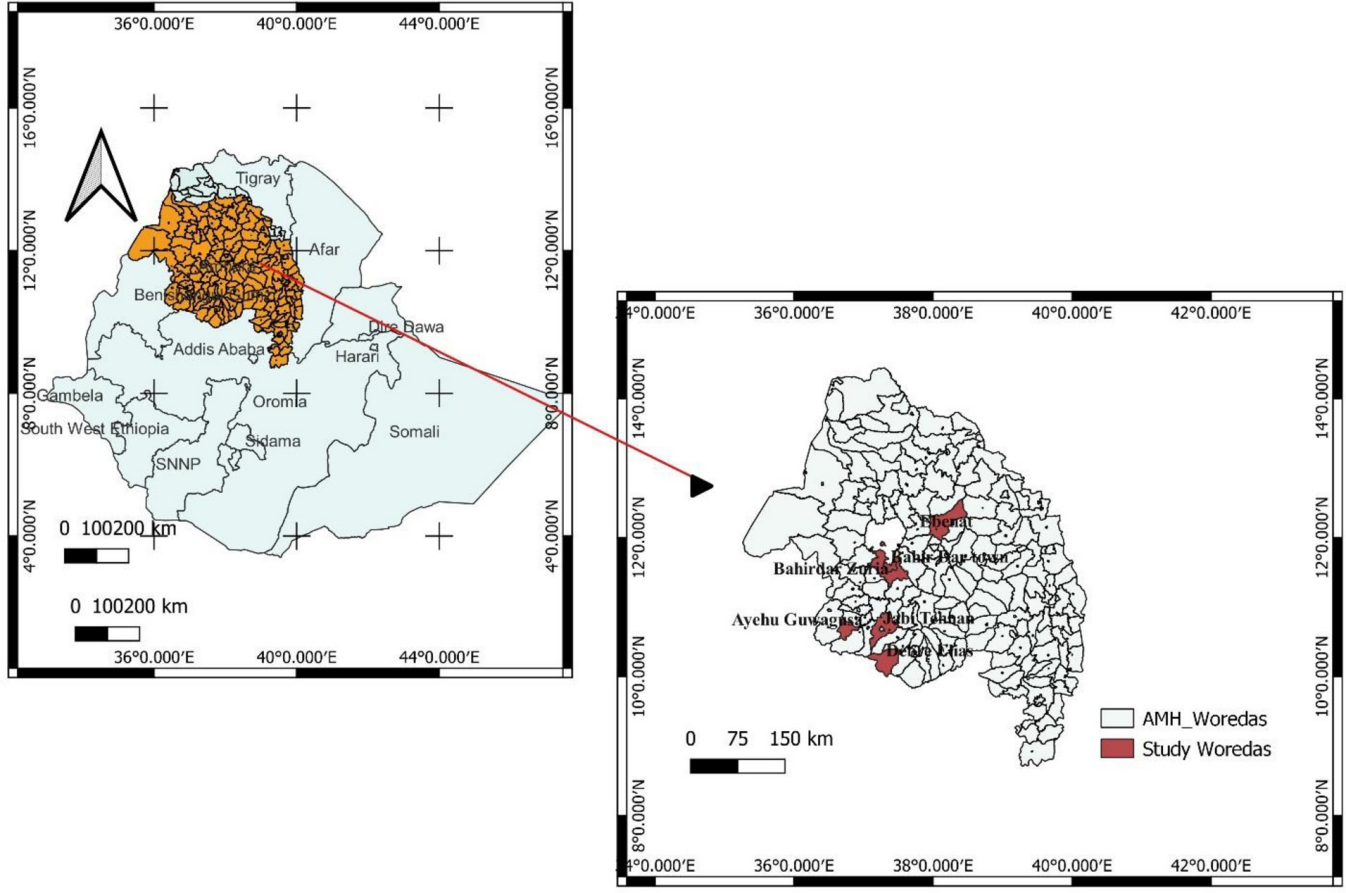
Map of the study districts where *Anopheles* mosquito sample collection were conducted in west Amhara Region, northwest Ethiopia.

A community- based cross-sectional study was conducted from three districts (Ayehu-Guagusa, Jabitehana and Ebinat) “Fig 1” of west Amhara Region from 08 February–30 March 2023. According to the Central Statstical Agency of Ethiopia “[20]”, Ayehu-Guagusa, had a total population of 229, 405, of whom 113,800 and 115,605, were males and females. Jabitehnan district had a total population of 205,076, of whom 101, 983 and 103, 093 were males and females. Ebinat district had a total population of 250,452, of whom 126,888 and 123,564 were males and females. These three districts also vary in altitude ranging from < 1500 meters above sea level (asl) in Ayehu-Guagusa district to 1500 and 2500 meters asl in Jabitehnan and Ebinat districts. Although malaria transmission is seasonal (have major and minor transmission seasons) in the three selected districts, the region has a drastic altitudinal range from 506 meters at the Blue Nile Gorge to 4533 meters at Ras Dejen mountain that create favorable environment for malaria vectors breeding “[10]”.

### *Anopheles* mosquito collection methods

Since the sample collection was conducted in minor malaria transmission season, three districts were selected based on high malaria case report. Then, five kebeles (sub-districts) from each district were selected for *Anopheles* mosquito collection. Before *An*. mosquito sample collection, potential breeding sites were inspected in collaboration with sub-district health extension workers. Then, *An*. mosquito collection was conducted roughly an average of 350 meters from the breeding sites using Centers for Disease Prevention and Control (CDC) light traps. Then, *An*. mosquito collection was conducted only one night from each selected sub-districts. (i.e 6 CDC light traps (3 indoor (bedrooms and dining rooms) and 3 outdoor (cow shelter, goat shelter, pit latrine etc.) were set for one night from 18:00 to 6:00 hrs.).

### *Anopheles* mosquito species identification

In brief, immediately after collection, mosquitoes were killed using chloroform and female *Anopheles* mosquitoes were sorted and identified using standard morphological key (Coetzee, 2020) under the dissection microscope at field. In the meantime, their abdominal status was determined based on blood digestion and ovarian development using standard key “[21]” as unfed, freshly fed, half gravid and gravid. Finally, the collection date, feeding status, collection site, sub-district were labeled and individually stored in 1.5ml of Eppendorf tubes and transported to APHI Medical Entomology Reference Laboratory for further analysis.

### Circumsporozoite protein-ELISA

From all collected and identified female *An*. mosquito species, Circumsporozoite Protein, Enzyme-Linked Immunosorbent Assay (CSP-ELISA) was performed to detect the circumsporozoite surface protein (CSP) of *P*. *falciparum* with monoclonal antibody Pf2A10- 01, *P*. *vivax* 210 with monoclonal antibody VK-210, and *P*. *vivax* 247 with monoclonal antibody VK-247 in the head and thoracic portion of the female *An*. mosquitoes, according to the method of “[22]”. All CSP-ELISA-positive *An*. mosquitoes were further analyzed by boiling at 100°c for 10 minutes using heat block as described by “[23]”. In brief, for CSP test, the monoclonal antibodies were coated on ELISA plate and incubated for half an hour. The plates were aspirated and banged upside down on white tissue paper. A similar fashion was also done after which the wells were filled with 200 μl blocking buffer. Fifty microliters of samples, negative and positive controls were added and incubated for 2hrs. at room temperature. Then, 50μl peroxidase conjugate was added to the wells and incubated in the dark at room temperature. Finally, 100 μl of substrate was added and incubated for 30 min in the dark at room temperature; the color change was examined with a reader at 405-414nm. Positive and negative results were determined based on the manufacturer’s instructions.

### Determination of blood meal sources of *Anopheles* mosquitoes

At APHI, the blood meal origins of all freshly fed and half-gravid female *An*. mosquitoes (*An*. *demeilloni*, *An*. *cinereus*, *An*. *gambiae* complex, *An*. *pharoensis*, *An*. *coustani*, *An*. *pretoriensis*, and *An*. *squamosus*), were determined as human, bovine and goat blood sources using direct ELISA “[24]”. In brief, for blood meal origin test, each mosquito abdomen was grounded with 50μl of phosphate-buffered saline (PBS) using pestle. The pestle was rinsed twice with 225μl PBS to make a total of 500μl final volume, and 50μl homogenate was added to 96-well ELISA plates.

Similarly, 50μl human sera (1/100 in PBS) and50μl PBS alone were used as a positive and negative control, respectively. Then, the plates were covered and incubated at room temperature for 3hrs. After incubation, the well contents were discarded, and the plates were tapped upside-down five times on white tissue paper and washed three times with 200μl PBS-Tween-20 using ELISA washer. Then, 50μl human peroxidase conjugate was added; plates were covered and incubated for 1hrs in the dark at room temperature. After that, plates were washed with ELISA washer three times with 200μl PBS-Tween-20, and 100μl of ABTS was added to each well and incubated for 30 min in the dark for human blood detection. In the end, the color change was visualized against the controls, and the results were read at 405nm absorbance using an ELISA plate reader. Likewise, similar methods were performed for bovine and goats blood detection except the bovine and goat sera, bovine and goat peroxidase conjugate were used respectively for bovine and goat blood detection.

### Data analysis

Data were entered and cleared in excel and then exported to SPSS version20 and analyzed using descriptive statistics to present species composition, infection rates and feeding preferences of *Anopheles* mosquito species. At the end, p -value < 0.05 were considered as statistically significant.

### Ethical consideration

Ethical approval was obtained from the College of Medicine and Health Sciences, Bahir Dar University, Bahir Dar, Ethiopia. Permission letters were obtained from APHI and then from each zone department, district health office. Verbal consent was obtained from each *kebeles* (sub-districts) administration and the head of each households before data collection commenced.

## Results

In this study, 261 *An*. mosquitoes comprising seven species were collected and identified with the most abundant species, *An*. *demeilloni* accounting for 112 (42.9%). Our study also depicted indoor to outdoor resting preference and had statistically significance association (p < 0.001) “Table 1”.

**Table 1:** Species composition and resting behaviors of *Anopheles* mosquitoes collected at three districts of west Amhara Region, northwest Ethiopia, from 08/02/2023–30/03/2023.

Species identified	Resting behavior				
	Indoor	Outdoor	Total	Chi-square	p- value
	N (%)	N (%)	N (%)		
*An*. *demeilloni*	82 (31.4)	30 (11.5)	112 (42.9)		
*An*. *cinereus*	37 (14.2)	56 (21.5)	93 (35.6)		
*An*. *gambiae* complex	27 (10.3)	13 (5)	40 (15.5)		
*An*. *pharoensis*	3 (1.1)	1 (0.4)	4 (1.5)	28.9	< 0.001
*An*. *coustani*	4 (1.5)	1 (0.4)	5 (1.9)		
*An*.*pretoriensis*	1 (0.4)	2 (0.8)	3 (1.1)		
*An*.*squamosus*	1 (0.4)	3 (1.1)	4 (1.5)		
Total	155 (59.4)	106 (40.6)	261 (100)		

**Fig 2:**
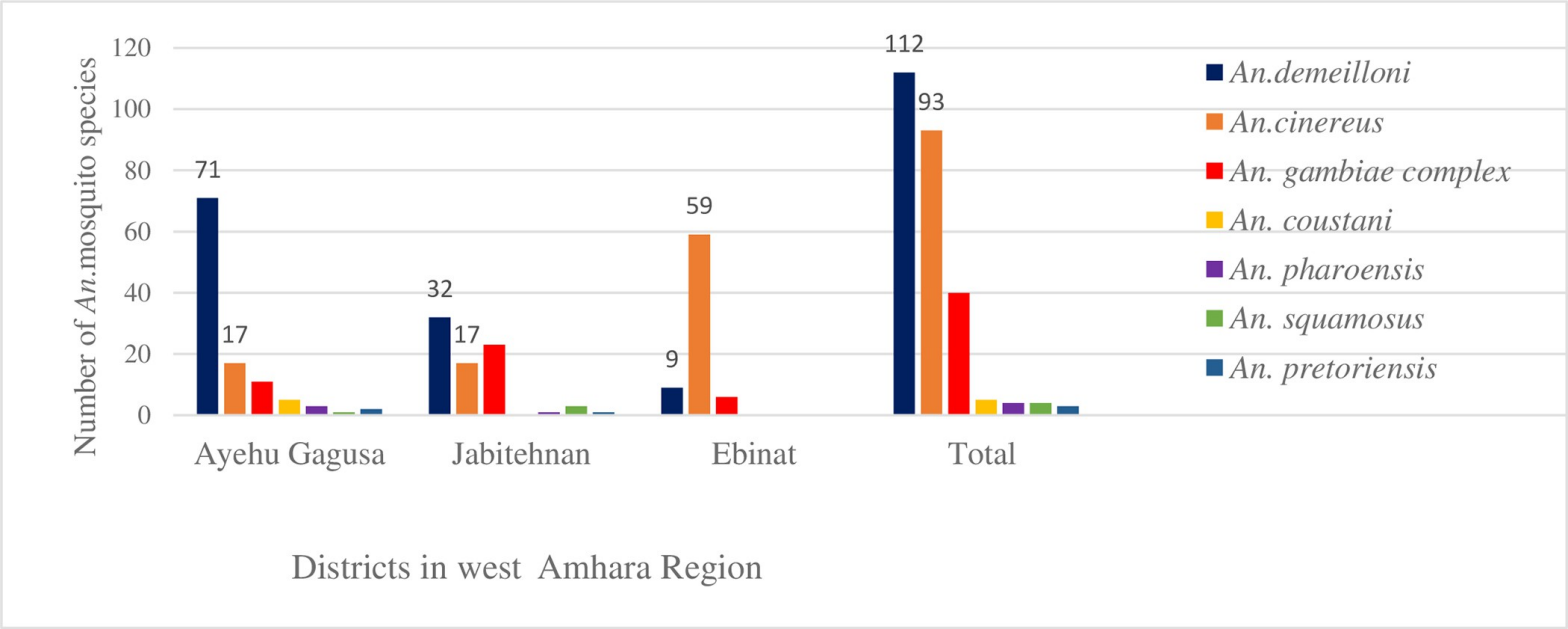
*Anopheles* mosquito species by districts, west Amhara Region, northwest Ethiopia, from 08/02/2023–30/03/2023.

*An*. mosquito species composition by district depicted that *An*. *demeilloni* was the most abundant species at Ayehu-Guagusa followed by Jabitehnan districts. While *An*. *cinereus* was, the most abundant species at Ebinat “Fig 2”.

Of the total 261 *An*. mosquito species tested for CSP-ELISA, 1 (0.4%) of *An*. *gambiae* complex was infected with *P*. *falciparum* parasite. While *An*. *demeilloni* and *An*. *cinereus* accounting for an overall infection rate of 3 (1.1%) by *P*. *vivax* parasite. Finally, mixed (*P*. *falciparum* and *P*. *vivax*) infections were not observed in our study “Table 2”.

**Table 2:** Infection rate of *Anopheles* mosquito species collected at three districts of west Amhara Region, northwest Ethiopia, from 08/02/2023–30/03/2023.

Species identified	Infection rate								
	CSP PF		Infection rate	CSP PV210		Infection rate	CSP PV247		Infection rate
	Positive	Negative		Negative	Positive		Negative	Positive	
*An*. *demeilloni*	112	0	0	111	1	0.4%	111	1	0.4%
*An*. *cinereus*	93	0	0	91	2	0.8%	91	2	0.8%
*An*. *gambiae* complex	39	1	0	40	0	0	40	0	0
*An*. *pharoensis*	4	0	0	4	0	0	4	0	0
*An*. *coustani*	5	0	0	5	0	0	5	0	0
*An*. *pretoriensis*	3	0	0	3	0	0	3	0	0
*An*. *squamosus*	4	0	0	4	0	0	4	0	0
Total	260	1	0.4%	258	3	1.1%	258	3	1.1%

Abdominal status of female *An*. mosquito species revealed that 121 (46.4%) of them were freshly fed and half-gravid. While unfed and gravid females altogether accounting for 140 (53.6%) of the total collected *An*. mosquitoes “Table 3”.

**Table 3:** Abdominal status of female *Anopheles* mosquito species collected at three districts of west Amhara Region, northwest Ethiopia from 08/02/2023–30/03/2023.

Species identified	Abdominal status of female *Anopheles* mosquito species						
	F*	G*	HG*	UF*	Total	Chi-square	p- value
	N (%)	N (%)	N (%)	N (%)	N (%)		
*An*. *demeilloni*	6 (2.3)	2 (0.8)	4 (1.5)	43 (14.5)	112 (42.9)		
*An*. *cinereus*	19 (7.3)	7 (2.7)	6 (2.3)	61 (23.4)	93 (35.6)		
*An*. *gambiae* complex	15 (5.7)	2 (0.8)	2 (0.8)	21 (8)	40 (15.3)		
*An*. *pharoensis*	2 (0.8)	0	0	0	2 (0.8)	28.9	< 0.002
*An*. *coustani*	3 (1.1)	0	0	2 (0.8)	5 (1.9)		
*An*. *pretoriensis*	3 (1.1)	0	0	0	3 (1.1)		
An. squamosus	4 (1.5)	0	0	0	4 (1.5)		
Total	109 (41.8)	11 (4.2)	12(4.6)	129 (49.2)	261 (100)		

Key: *F, G, HG, UF = Fed, Gravid, Half gravid and unfed

Of the 121 freshly fed and half-gravid *An*. mosquito species tested using blood meal-ELISA, mixed blood meal sources were not observed. However, 68 (56.2%) of them were positive for cattle blood only. On the contrary, 53 (43.8%) of them fed on unidentified host(s) (host (s) not identified using anti-human, anti-cattle and anti-goat antibodies) “Table 4”.

**Table 4:** Overall blood meal sources of *Anopheles* mosquito species collected at three districts of west Amhara Region, northwest Ethiopia from 08/02/2023–30/03/2023.

Species identified	Feeding preference status											
	Human blood only				Bovine blood only				Goat blood only			
	Negative	Positive	Total	%	Negative	Positive	Total	%	Negative	Positive	Total	%
*An*.*demeilloni*	67	0	67	0	24	43	67	35.5	67	0	67	0
*An*. *cinereus*	25	0	25	0	10	15	25	12.4	25	0	25	0
*An*. *gambiae* complex	17	0	17	0	12	5	17	4.1	17	0	17	0
*An*.*pharoensis*	2	0	2	0	1	1	2	0.8	2	0	2	0
*An*. *coustani*	3	0	3	0	2	1	3	0.8	3	0	3	0
*An*.*pretoriensis*	3	0	3	0	1	2	3	1.7	3	0	3	0
*An*. *squamosus*	4	0	4	0	3	1	4	0.8	4	0	4	0
Total	121	0	121	0	53	68	121	56.2	121	0	121	0

## Discussion

Reports showed that Amhara Region is one of the most malaria prevalent region in Ethiopia. Unpublished data from the APHI also depicted that there is an increment in the prevalence of malaria in the Amhara Region of northwest Ethiopia, though vector control tools have been implemented. One of the possible reason might be due to lack of comprehensive information on the currently existing *An*. mosquito species composition, infection rate and feeding preferences.

In this study, *An*. *demeilloni* was the overall most abundant species collected among all captures and accounting for 42.9%. This species is documented in Ethiopia, in an updated list of *An*. mosquito species in the Afrotropical Region in 2020 “[25]”, even if it was not considered as a primary or secondary malaria vector. This finding was higher than other previous reports by “[15]” in the Jabitehnan district of northwest Ethiopia, “[14]” at Ghibe River Basin in southwest Ethiopia, [11]” in high and low land parts of Derashe district southern Ethiopia, “[13]” in high land part of Derashe district southern Ethiopia. The difference might be due to the difference in sample collection period. In all other studies, *An*. mosquito collection was conducted at least for a year (including both major and minor malaria transmission seasons) and hence large numbers of *An*. mosquito species were collected and the percentage of *An*. *demeilloni* became smaller compared to our study.

The present study also depicted that *An*. *demeilloni* was the predominant indoor site species accounting for 31.4% of all captures. This might be due to the strong endophillic and endophagic behavior of this species or it might be due to the strong tendency of this species attracted by light and hence trapped by CDC light traps at indoor sites. This finding was higher than reports by “[14]” in the Ghibe River Basin in southwest Ethiopia where the indoor collection was 4.2%. This difference might be due to the difference in the number of collected adult female mosquitoes (261 vs 1801) or it might be due to the change in feeding and resting behavior of this species depending on the availability of the host.

On the other hand, *An*. *cinereus* was the predominant outdoor site species accounting for 21.5% of all captures. This was in harmony with the study conducted by “[14]” in Ghibe River Basin southwest Ethiopia where larger numbers of *An*. *cinereus* were collected at outdoor sites.

Composition of *An*. mosquito species by district revealed that *An*. *demeilloni* was the most abundant at Ayehu-Guagusa followed by Jabitehnan districts. On the contrary, *An*. *cinereus* was the predominant species at Ebinat district. This variation might be due to altitudinal variations that resulted in variations of temperature and humidity “[3, 13]”.

Our study showed that the overall infection rate of *An*. *demeilloni* and *An*. *cinereus* was 1.1% with *P*. *vivax* parasite. This incriminates *An*. *demeilloni* and *An*. *cinereus* as potential vectors of malaria at the study areas. This finding was different from previous studies conducted by “[14]” where *An*. *demeilloni* and *An*. *cinereus* were negative for CSP of *P*. *falciparum* and *P*. *vivax* parasites in the Ghibe River Basin in southwest Ethiopia. In addition, “[13]”where 0.64% of *An*. *demeilloni* was positive with a CSP of *P*. *falciparum* and negative for CSP of *P*. *vivax* parasites in the high land of Derashe district southern Ethiopia.

In this study, large proportion (49.2%) of *An*. mosquito species collected were unfed. This finding was in harmony with studies conducted by “[17]” in Burie district, West Gojjam Zone, Amhara Region, and “[14]” in Ghibe River basin Southwest Ethiopia where large proportion of unfed *An*. mosquitoes were collected using CDC light traps. Large proportion of unfed *An*. mosquitoes collected in our study could be due the fact that unfed *An*. mosquitoes were caught during searching of their blood meal before they took blood. However, reports by “[15]”, at Jabitehnan district, West Gojjam Zone, Amhara Region, reported contrary findings. This could be due to sharing of same houses by human and other domestic animals and hence, creating additional and alternative blood meal sources for *An*. mosquitoes “[18]”.

In the current study, though none of the freshly fed and half-gravid *An*. mosquito species fed on humans and goats, the majority of blood meal sources were cattle blood accounting for 56.2%. However, “[17, 26]” and “[19]”, reported different results. The possible reason for this might be due to difference in host selection behavior, flight range and blood feeding patterns of each *An*. mosquito species “[27]”.

Our study depicted that among all freshly fed and half-gravid *An*. mosquito species, none and 35.5% of *An*. *demeilloni* fed on human and cattle, respectively. While “[14, 26]”, reported different findings, where, none of the *An*. *demeilloni* species fed both human and cattle and 3.6% and 58.5% of them fed on human and cattle, respectively. The possible reason for this might be due to difference in host availability and shifting of feeding behaviors of each *An*. mosquito species “[28]”. Finally, sinc**e**, our study used cross-sectional study design with a short period and was conducted in minor malaria transmission season; this might affect the inference of the results.

## Conclusions

In this study, about 60% of the overall *An*. mosquito species collected prefer indoor to outdoor resting behaviors. *An*. *demeilloni* showed the most abundant species among all captures. In addition, *An*.*demeilloni* and *An*.*cinereus* revealed variations among districts. Moreover, *An*. *demeilloni* and *An*. *cinereus* might be considered as potential malaria vectors in the study areas. Furthermore, almost 50% of the *An*. mosquito species collected were unfed and more than 50% of the freshly fed and half-gravid *An*. mosquito species collected fed on cattle blood implying shifting of feeding behaviors. As a result, further longitudinal studies on species composition, infection rates and feeding preference of should be done in the future by including more areas, incorporating more antibodies.

## Supporting information

S1 File All relevant data are within the manuscript and its supporting information files. (XLSX)
